# Feeding in Gastric Ulcer

**Published:** 1902-03-15

**Authors:** 


### FEEDING IN GASTRIC ULCER.

Often enough the medical attendant finds much difficulty in arriving at a suitable dietary in the treatment of gastric ulcer; the indications are plainly to administer food only in such form as will not irritate the heating ulcer, but in practice this desirable end is not seldom a hard problem. Of course the difficulties may be obviated, for a time, by rectal feeding, but this process cannot be continued for any lengthy period without impairment to the general health of the patient. Sir Lauder Brunton's recent clinical lecture[1] on "Feeding in Gastric Ulcer" contains many invaluable practical hints which it is well to bear in mind. That such hard, indigestible or irritating substances as seeds, the achenia of strawberries, nuts, butcher meat, currants, pepper, etc., must be avoided is well recognised by medical practitioners, and reliance is placed chiefly on fluid foods. But it is not always sufficiently recognised that even such a simple fluid food as milk may result in the formation of hard indigestible coagula in the stomach. Brunton relates an instance where a female patient vomited a hard felt-like concretion, and another case where a similar fibrous concretion was removed per rectum, and in each case the concretion was formed of long curds of casein matted together, being the result of the rapid ingestion of milk. Hence the advantage of adding lime water to the milk, or of peptonising it so as to avoid those large indigestible curds being formed.

The next point of great practical moment which Brunton emphasises is the fact that the longer food remains in the stomach the more acid does it become. We should therefore be careful to administer the food in small quantities at frequent intervals, rather than in three or four meals of larger quantity.

Further, the greater ease with which the digestion of *pounded* fish or meat as compared with the small solid masses brought about by mincing or mastication is a point to remember when a more extended diet is to be allowed.

Lastly, the proper way of preparing starchy food is touched upon, and we are reminded that we should beat the starch up with a little cold water, and then, when it is a paste, hot water may be added without causing lumpiness, while no one should be ignorant of the indigestibility of new bread as compared with stale.

[1] Brit. Med. Journ. Mar. 1, 1902.

